# Unexpected observation of spatially separated Kondo scattering and ferromagnetism in Ta alloyed anatase TiO_2_ thin films

**DOI:** 10.1038/srep13011

**Published:** 2015-08-12

**Authors:** T. P. Sarkar, K. Gopinadhan, M. Motapothula, S. Saha, Z. Huang, S. Dhar, A. Patra, W. M. Lu, F. Telesio, I. Pallecchi, Ariando, D. Marré, T. Venkatesan

**Affiliations:** 1 Department of Physics, National University of Singapore, Singapore 117542; 2 NUSNNI-NanoCore, National University of Singapore, Singapore 117576; 3 Department of Electrical and Computer Engineering, National University of Singapore, Singapore 117576; 4 CNR-SPIN and Università di Genova, Dipartimento di Fisica via Dodecaneso 33, Genova Italy 16146

## Abstract

We report the observation of spatially separated Kondo scattering and ferromagnetism in anatase Ta_0.06_Ti_0.94_O_2_ thin films as a function of thickness (10–200 nm). The Kondo behavior observed in thicker films is suppressed on decreasing thickness and vanishes below ~25 nm. In 200 nm film, transport data could be fitted to a renormalization group theory for Kondo scattering though the carrier density in this system is lower by two orders of magnitude, the magnetic entity concentration is larger by a similar magnitude and there is strong electronic correlation compared to a conventional system such as Cu with magnetic impurities. However, ferromagnetism is observed at all thicknesses with magnetic moment per unit thickness decreasing beyond 10 nm film thickness. The simultaneous presence of Kondo and ferromagnetism is explained by the spatial variation of defects from the interface to surface which results in a dominantly ferromagnetic region closer to substrate-film interface while the Kondo scattering is dominant near the surface and decreasing towards the interface. This material system enables us to study the effect of neighboring presence of two competing magnetic phenomena and the possibility for tuning them.

There has been an upsurge of interest in Kondo scattering and ferromagnetic systems investigating cooperation between these two opposing effects^1^,^2^ and also exotic effects such as weak ferromagnetism and Kondo screening in Kondo lattice systems^3^,^4^,^5^. The Kondo effect is one of the well-known and widely studied phenomena in condensed matter physics where a dilute magnetic impurity in a conducting sea introduces a resonant scattering which results in an increase of resistivity below a characteristic temperature called the Kondo temperature (*T*_*K*_)^1^,^2^,^6^. The conduction electrons scatter from the magnetic impurity via an antiferromagnetic interaction causing a spin flip in time scales of the order of femtosecond. The Kondo scattering is a sensitive measure of a magnetic impurity in a conductor.

Kondo scattering was first observed in anatase TiO_2_ thin films via Nb subtitution^7^. Within a short period, ferromagnetism was demonstrated in Ta alloyed anatase TiO_2_ thin films^8^. Hence one can use Kondo scattering as a litmus test for when a dopant becomes a magnetic impurity in a host. If higher concentration of such magnetic centers can be created and if the magnetic centers are interacting, one could possibly achieve ferromagnetism in such host materials.

Since the interactions are opposite, one does not expect Kondo effect and ferromagnetism to coexist in the same system. However, there have been reports of simultaneous observation of Kondo scattering and ferromagnetism in heavy fermion systems (which include lanthanides and actinides) with partially filled 4f or 5f shells^3^,^4^,^5^,^9^,^10^,^11^,^12^, which can be theoretically modeled as a regular lattice of local moments coupled to conduction electrons. Can such effects be seen in simple 3d electron systems?

The Kondo effect is the result of an antiferromagnetic exchange interaction with antiparallel alignment of spins between conduction electrons and the magnetic ion. This is opposite to ferromagnetism where the itinerant electrons mediate and stabilize an exchange interaction with a parallel alignment of spins between magnetic ions. However, the Kondo effect is a sensitive electrical manifestation of the presence of localized magnetic moments in a system. Wide band gap semiconductors, like TiO_2_, involve spin, orbital and charge degrees of freedom to produce local magnetism thus making them promising for observation of Kondo phenomena. Several groups have observed this effect in diluted magnetic impurity doped TiO_2_, like Ti_1–x_Co_x_O_2_^13^. Even non-magnetic transition element such as Nb in TiO_2_ has shown Kondo phenomena where the magnetic centers were attributed to the Ti-vacancy, as reported by Zhang *et al*.^7^. Recently, ferromagnetism has been observed^14^ in Nb doped TiO_2_ film at room temperature which has been attributed to the presence of Ti^3+^. Ferromagnetism and the possibility of controlling both the spin and charge degrees of freedom in these dilute magnetic semiconductors (e.g. TiO_2_^13^,^14^, In_2_O_3_^15^, SnO_2_^16^, ZnO^17^) have received much attention in recent past for future spintronic devices at room temperature. Our current understanding of the magnetism in this system is either carrier mediated Ruderman-Kittel-Kasuya-Yosida (RKKY) interaction between the magnetic centers which were identified as Ti-vacancies^8^ or through magnetic polarons or possibly a Stoner exchange model. Further detailed experiments are needed to distinguish between these models.

Here we report on the observation of ferromagnetism and Kondo scattering in anatase Ta_0.06_Ti_0.94_O_2_ thin films as a function of thickness (10–200 nm) grown on SrTiO_3_ (100). We observe spatially separated ferromagnetism and Kondo scattering in the same system and by studying the distribution of the defects, magnetism and transport in the films as a function of thickness, we are able to reconcile the observation. The anatase Ta_0.06_Ti_0.94_O_2_ thin films system is inferred to be an exciting candidate for elucidating ferromagnetism and Kondo scattering in a highly correlated electronic system with the possibility of tuning them.

## Results and Discussion

**Figure 1:**
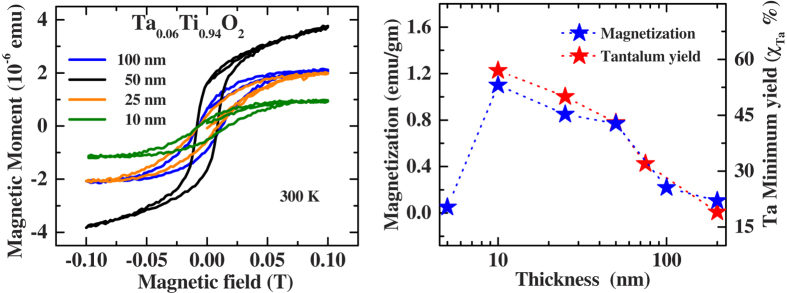
Magnetization and Ta minimum yield as a function of thickness. (**a**) Magnetization (M) vs. applied magnetic field (H) characteristics of Ta:TiO_2_ as a function of thickness. (**b**) Magnetization (M) and tantalum channelling minimum yield (χ) behavior as a function of thickness. All these measurements were performed at room temperature.

Recently, we have shown that incorporation of Ta in anatase TiO_2_ introduces magnetism which peaks at about 6% Ta concentration^18^. In this paper, we chose Ta-6%:TiO_2_ and studied the magnetism as a function of thickness. Figure 1(a) shows the typical in-plane M-H hysteresis loop of Ta:TiO_2_ at 300 K as a function of thickness. The substrate and the undoped TiO_2_ is purely diamagnetic (Fig. S1) compared to a Ta:TiO_2_ film. In 5 nm thick film, no magnetism could be observed while ferromagnetism appears at a thickness of ~10 nm and the magnetization decreases monotonically with increasing thickness, as shown in Fig. 1(b).

**Figure 2:**
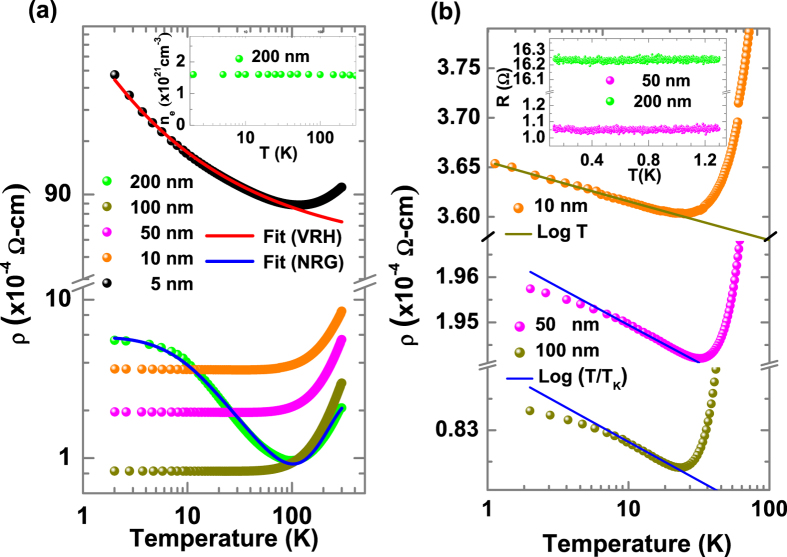
Temperature dependent resistivity as a function of film thickness and carrier density. (**a**) Temperature dependent resistivity (ρ) as a function of thickness along with the variable range hopping (VRH) fit for a film of thickness 5 nm and numerical renormalization group (NRG) theory fit for a thickness of 200 nm. Inset shows the carrier density (n_e_) as a function of temperature (T). (**b**) Resistivity (ρ) as a function of temperature for 10, 50 and 100 nm films and numerical renormalization group (NRG) theory fit of the low temperature part. Inset shows the resistance (R) vs. temperature (T) characteristics of 200 and 50 nm samples at very low temperatures.

Figure 2(a) shows the resistivity (ρ) vs. temperature (T) for films of different thicknesses. The resistivity of all the samples is of the order of 10^−4^ Ω-cm whereas that of 5 nm film is orders of magnitude higher. The 5 nm film undergoes crossover from metallic to strongly localized behavior as the temperature decreases below 100 K. The strong localization regime has the character of 2D variable range hopping (VRH) as the ρ vs. T data fits well with theoretical formula for conductivity σ = C exp-(T_0_/T)^1/3^ from 2 to 100 K, where C and T_0_ are constants. It is to be noted that the 5 nm film does not exhibit any magnetism (below the detection limit of the SQUID). The 5 nm film has poor conductivity which does not allow any kind of exchange interaction involving carriers. This result argues against a polaronic picture for the ferromagnetism in this system. Interestingly, the 10 nm thick film also exhibits localization at low temperatures which follows a *logT* behavior in the temperature range of 2 to 35 K, as shown in Fig. 2(b). As the thickness is further increased the 50 nm film also undergoes a crossover from a metal to a kind of localization-like behavior as shown in Fig. 2(b), which cannot be fitted with the *logT* relation at very low temperatures. This temperature where the *logT* behavior deviates increases in 100 nm and 200 nm films, as shown in Fig. 2(b).

The resistivity of 50 and 100 nm thick films saturates at very low temperatures below 1.3 K. There is an uncertainty in the saturation temperature of these samples in the temperature range from 2 to 1.3 K as we could not obtain the resistivity data due to change in the measurement system (going from PPMS to dilution refrigerator). Hence we fitted the low temperature resistivity data with a *log(T/T*_*K*_) term rather than numerical renormalization group (NRG) formula because NRG formulation requires saturation of resistivity. However, the 200 nm film saturates at temperatures above 2 K (Fig. 2a). The entire resistivity behavior of the 200 nm thick film (which shows negligible magnetization) was fitted with the NRG Kondo relation:


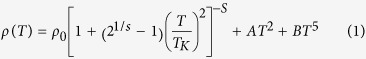


where *T*_*K*_ (=15.5 K) is the Kondo temperature and *s* (=0.43) is the spin parameter, *ρ*_0_ (=5.87 × 10^−4^ Ω-cm) is the zero temperature resistivity, A (2.67 × 10^−9^) & B (2.45 × 10^−17^) are the fitting parameters. A Kondo system with a Kondo temperature *T*_*K*_ can be approximated for resistivity as *ρ*_0_ − *cT*^2^ (for 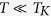) and *logT* (for *T* > *T*_*K*_) where *ρ*_0_ is the residual resistivity. Alternatively, roughly middle of the logarithmic region (*logT* behavior) is taken as the Kondo temperature. In the thermal conductivity or heat capacity measurements, *T*_*K*_ appears as a maximum in the temperature plot due to a change in the entropy of the system. By definition, minimum in the resistivity is not taken as the Kondo temperature as the minimum is a result of the competition between Kondo scattering (due to spin-flip process) and non-Kondo scattering processes involving electron-phonon and electron-electron.

The point to be noted is that in the Kondo temperature regime there are only two fitting parameters (*s* and *T*_*K*_) and the A (electron-electron scattering) and B (electron-phonon scattering) terms are fits to the high temperature part of the data. We included the electron - electron interaction term (*AT*^*2*^) which arises at high temperatures probably due to correlation effect, as the phonon term alone cannot explain the high temperature resistivity evolution. The Hall measurement shows that the carrier concentration is independent of temperature as shown in the inset of Fig. 2(a). So, these observations ascertain a Kondo-like transport. Another proof for Kondo scattering is the existence of a peak in the thermopower measurement close to the measured Kondo temperature (Supplementary, Fig. S2). A further proof of Kondo behavior is the saturation of susceptibility at low temperatures (Supplementary, Fig. S3) as predicted by Rajan^19^. The 50 and 100 nm thick films also reveal Kondo-like behavior, and their resistivities do saturate at very low temperatures (Inset of Fig. 2(b)). These films exhibit much larger magnetization as compared to the 200 nm film. The magnetoresistance measured in these films shows interesting behavior but nevertheless is isotropic as expected for Kondo scattering. We will address these interesting observations later in the paper.

However the key question to ask here is how come the original single impurity Kondo model developed for metallic systems is still valid for the oxides? There are three major differences- the carrier density for metals is 3 orders of magnitude larger, the magnetic impurities for metals are in the range of 0.001% while for the case of oxides it is ~2–3% and the metals like copper have no correlation effects unlike the oxides. The established understanding is that above a characteristic Kondo temperature *T*_*K*_, the interaction of the impurity spin with the conduction electron spin is very weak, but at temperatures much lower than *T*_*K*_, the impurity spin is completely screened by the conduction electrons. The radius of the screening cloud is predicted to be of the order 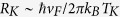
20. For example, in the metallic system AuFe with a *T*_*K*_ of 0.3 K, *R*_*K*_ is ~3 μm^21^. However in the case of oxides, the Fermi velocity *v*_*F*_ is much smaller than metals due to lower carrier concentrations and the observed Kondo temperature seems much higher. This leads to a small sized electron cloud (smaller by at least two orders of magnitude) which may lead to a reduced net average spin polarization than metals. While this will allow for a larger concentration of magnetic impurities without overlap of the Kondo electron cloud (thereby allowing application of the single impurity Kondo theory) the reduced average spin polarization of the Kondo cloud for oxides may not give rise to the expected scattering. Add to this strong correlation effects which are yet to be modeled theoretically and it is indeed such a surprise that the NRG fit works so well for the 200 nm Ta:TiO_2_ film!

**Figure 3:**
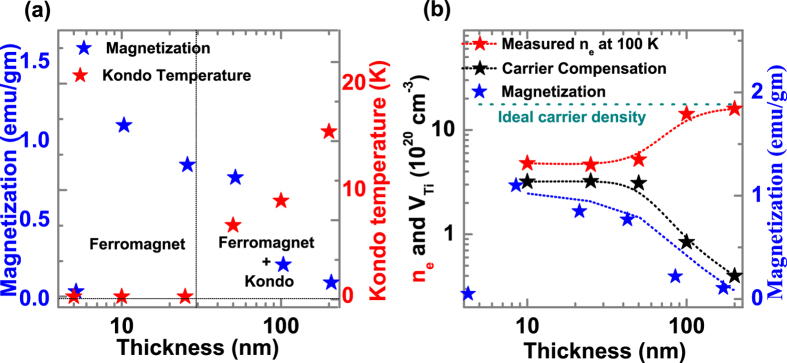
Variation of magnetization, Kondo temperature and carrier density with film thickness. (**a**) Magnetization (red asterisk) and Kondo temperature (blue asterisk) as a function of thickness. (**b**) The measured carried density (red asterisk), compensated carriers (black asterisk) and magnetization (blue asterisk) as a function of thickness.

Figure 3(a) shows two different regimes of film thicknesses where in one regime only ferromagnetism is observed while in the other both ferromagnetism and Kondo scattering are seen. As shown in Fig. 3(b), the magnetization decreases with increasing thickness whereas strength of the Kondo interaction (Kondo Temperature) increases. Films of intermediate thickness (~50 nm) show both Kondo and ferromagnetic behavior. The compensated carriers (i.e. the difference between the ideal carrier density and the measured carrier density) follow a similar behavior to the normalized magnetic moment as a function of thickness (Fig. 3(b)). This similarity in the thickness dependence is because the carrier compensation arises from Ti-vacancies (V_Ti_) that are also responsible for the observed magnetization^8^.

**Figure 4:**
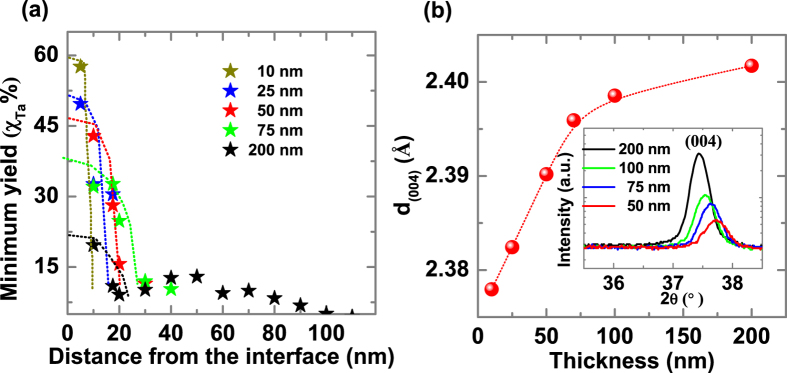
Variation of Ta channeling minimum yield and lattice spacing d_(004)_ with film thickness. (**a**) Tantalum channelling minimum yield (χ) with the depth varying from the interface to the surface as a function of thickness. (**b**) Lattice spacing (d) estimated from (004) XRD peak as a function of thickness. Inset shows the evolution of (004) XRD peak position with change in film thickness.

The correlation between the defects and magnetization is experimentally determined using Rutherford back scattering (RBS)/ channeling. RBS channeling data show depth dependent minimum yield of Ta atoms (proportional to the number of Ta ions misplaced from the regular atomic sites) from the interface as a function of thickness given in Fig. 4(a). Tantalum minimum yield in thinner film at the interface is higher than in thicker films. This could be due to defects which is higher near the interface in thinner films due to strain. Figure 4(b) shows d_004_-spacing of the film as function of thickness. The films are grown on STO (100) substrate which has higher lattice constant (3.90 ǻ) than TiO_2_ (3.79 ǻ) in the *ab*-plane. As a result, the *ab-*plane is under tensile strain on STO film which compresses the *c*-axis. As the film thickness increases, the strain relaxes thereby increasing the atomic-spacing along the *c*-axis as seen in the X-ray data. Sustaining a large number of cationic defects like vacancies requires interface strain. Hence when the strain is relaxed the number of defects in the film decreases.

In a prior study, we have shown that the origin of the ferromagnetism in Ta:TiO_2_ is from V_Ti_ with a minor contribution from oxygen site^8^. Tantalum 5+ valence element substitutes into the Ti site releasing an electron. As the carrier concentration increases with Ta concentration the formation energy for compensating defects such as V_Ti_ and Ti^3+^ decreases, with our film preparation condition favouring V_Ti_ formation as shown via X-ray absorption spectroscopy (XAS) and X-ray magnetic circular dichroism (XMCD) studies^8^. Since defects (predominantly V_Ti_) are responsible for the ferromagnetism this explains why the magnetization decreases with thickness. We have shown using photoluminescence that the oxygen vacancy related luminescent signal progressively disappears with increasing Ta concentration (Supplementary Fig. S4) and thus the predominant amount of defects are cationic, consistent with other measurements^22^. It should be noted that the magnetic moments are mainly arising from Ti-vacancies and as the film is thicker, the concentration of these moments reduces drastically, limiting the possibility of exchange interaction between them. We speculate that the same Ti-vacancies in the dilute limit are responsible for the Kondo effect as demonstrated for the case of Kondo effect in Nb substituted TiO_2_^7^.

**Figure 5:**
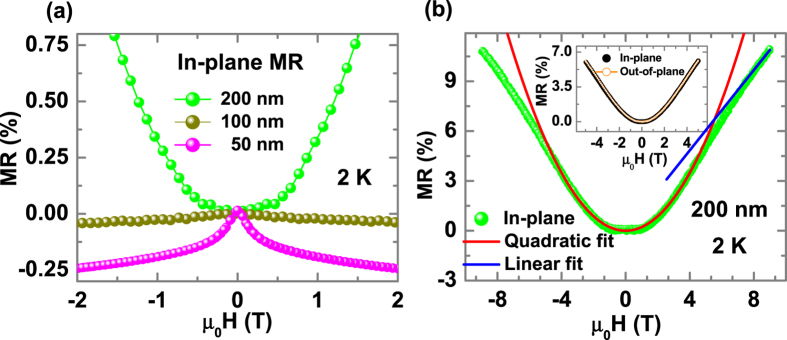
In and out-of-plane magnetoresistance as a function of thickness at 2 K. (**a**) In-plane magnetoresistance (MR) as a function of thickness. (**b**) In-plane magnetoresistance (MR) for 200 nm film along with the theoretical quadratic (red solid line) and liner fits (blue solid line). Inset shows the in-plane and out-of-plane magnetoresistance suggesting the absence of anisotropy.

In the samples of thickness 10–100 nm, the ferromagnetism is dominantly near the film-substrate interface while the Kondo scattering is contributed mainly by transport near the surface region. Then at low thickness both effects are spatially neighboring each other leading to the most interesting case of this study. However, for the thinner films the defect density at the surface is significant enough to weaken the Kondo scattering. With optimized film processing it may be possible to have both effects strong and in close proximity. In the 200 nm film the ferromagnetism is too small as the V_Ti_ density at the interface and the surface defect density are reduced and one sees nearly perfect Kondo scattering fit. Another interesting consequence of this interaction is the behavior of the magnetoresistance which is shown in Fig. 5(a,b). Figure 5(a) shows in-plane magnetoresistance (MR measured at 2 K) of 50, 100 and 200 nm film where the 50 nm and 100 nm films show a diverging negative magnetoresistance. The divergent nature of the MR confirms the existence of ferromagnetism in the films. The MR changes its sign from negative to positive with increasing thickness. The in-plane MR (current is parallel to magnetic field) and out-of-plane MR (current is perpendicular to magnetic field) in the 200 nm film is isotropic. The isotropy of the MR of the 200 nm film (inset of Fig. 5(b)) further confirms the Kondo effect. The positive magnetoresistance could be explained possibly in terms of the formation of a Kondo lattice which requires interactions between a Kondo system and a ferromagnetic region or a disordered lattice.

A Kondo-lattice features a competition between the Kondo effect and RKKY exchange interaction which affects the behavior of the MR. Positive MR could be an intrinsic effect of Kondo lattice in a strongly correlated oxide or heavily disorder system^23^,^24^,^25^,^26^. Ohkawa^27^ has reported that in an impurity Anderson model with infinitely large correlation effect (leading to a Kondo lattice), a positively linear MR can be observed for all fields for s = 1, while the MR may be quadratic at low fields (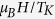 < 0.1) for spins of 0.4–0.6. Our measurement yields a *T*_*K*_ of 15.5 K, which implies a critical magnetic field (H) of 2.4 T below which the magnetoresistance is expected to show a quadratic behavior. To verify this, we fitted the magnetoresistance data with an expression A*H^*2*^ which is consistent with our data where we measure *s* = 0.43 from our experimental fit (Fig. 5(b)) with A (2.12 × 10^−7^), thus supporting the assumption of Kondo lattice. Above the critical field, the magnetoresistance behaves linearly with the applied magnetic field. A negative magnetoresistance is expected for 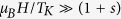, however our maximum applied magnetic field is only 9 T which is very low compared to the expected crossover field (~34 T). However, our resistivity vs. temperature at low temperatures does not show any signature of Kondo lattice formation. The absence of any ordered arrangement of the magnetic entities in the system argues against a Kondo lattice formation. On the other hand the presence of defects supports a disordered Griffith’s phase which also gives a positive magnetoresistance^28^. Manyala *et al*. have shown that disordered ferromagnetic system (Fe_1–y_Co_y_Si) can give isotropic positive magneto resistance^29^,^30^. So the observed positive magnetoresistance is most likely due to disordered Kondo centers (Griffith’s phase).

## Conclusions

We show that by incorporation of nonmagnetic element Ta in TiO_2_, one can induce ferromagnetism as well as Kondo scattering in the same system. As the magnetic ions originate from cationic defects and the defect density decreases from the substrate interface towards the surface this allows for the spatially separated ferromagnetism close to the interface and Kondo scattering closer to the surface. This may allow us to study exciting effects involving the interaction of ferromagnetism and Kondo scattering in a highly correlated electronic system such as disordered ferromagnets.

## Methods

### Sample preparation and measurements

Ta_0.06_Ti_0.94_O_2_ thin films were grown by pulsed laser deposition (PLD) on SrTiO_3_ (100) substrate at a temperature of 700 °C and at an oxygen partial pressure of 1 × 10^−5^ Torr. The PLD target was prepared by a solid-state reaction method using 99.999% pure TiO_2_ and Ta_2_O_5_ powders. The crystalline phase was determined by Bruker X-ray diffraction (XRD) which showed oriented growth of anatase phase along (004) direction. The Ta concentration and the thickness of the films were determined by Rutherford backscattering spectrometry (RBS). Moreover, ion channeling measurements indicated that Ti is fully substituted by Ta atoms in thick films closer to the surface (low minimum yield <3%) with increasing defects towards the substrate interface (higher minimum yields >10%). Secondary ion mass spectroscopy (SIMS) showed no magnetic impurities in the films or the substrate. Temperature dependent resistivity measurement was done in linear four probe geometry. Also both in-plane (magnetic field parallel to current) and out-of-plane (magnetic field normal to current) magnetoresistance (MR) measurements were carried out in linear four point geometry. Hall coefficient and carrier concentration were determined by applying a magnetic field (H) perpendicular to the film plane in the van der Pauw geometry (with Al wire bonding) in a physical property measurement system equipped with a 9 T superconducting magnet. The magnetic field was swept from 9 to −9 T under a constant current for both the above measurements. Seebeck effect was measured in a in a Quantum Design PPMS system equipped with thermal transport option, as a function of temperature from 10 to 300 K. We applied a square-wave heat flow along the film plane, with adjustable period (from 400 to 1450s) and thermal gradient (from 0.1 to few K), and measured the related voltage drop decay. The measurements were carried out both in zero field and in a magnetic field of 9 T, but no appreciable dependence on the magnetic field was observed.

## Additional Information

**How to cite this article**: Sarkar, T. P. *et al*. Unexpected observation of spatially separated Kondo scattering and ferromagnetism in Ta alloyed anatase TiO_2_ thin films. *Sci. Rep*. **5**, 13011; doi: 10.1038/srep13011 (2015).

## Supplementary Material

Supplementary Information
