# Supplementary material for: Unexpected observation of spatially separated Kondo scattering and ferromagnetism in Ta alloyed anatase TiO2 thin films
Source: Sci Rep. 2015 Aug 12;5:13011. doi: 10.1038/srep13011 (PMC4533010; doi:10.1038/srep13011)
Supplement: Supplementary Information [file srep13011-s1.pdf]

Supplementary

Unexpected observation of spatially separated Kondo scattering and ferromagnetism in Ta alloyed anatase TiO_2 thin films

T. P. Sarkar[1,2], K. Gopinadhan[2], M. Motapothula[2], S. Saha[2], Z. Huang[2], S. Dhar[2,†], A. Patra[2], W. M. Lu[2], F. Telesio[4], I. Pallecchi[4], Ariando[1,2], D. Marré[4], T. Venkatesan[1,2,3,*]

[1]Department of Physics, National University of Singapore, Singapore 117542

[2]NUSNNI-NanoCore, National University of Singapore, Singapore 117576

[3]Department of Electrical and Computer Engineering, National University of Singapore, Singapore 117576

[4]CNR-SPIN and Università di Genova, Dipartimento di Fisica via Dodecaneso 33, Genova Italy 16146

*Corresponding author: email: venky@nus.edu.sg, Phone: +65-65165187

[†]Present address: Department of Physics, Shiv Nadar University UP, India 201314

Additional results from Ta:TiO_2 samples

1. Magnetic moment vs. applied magnetic field characteristics for undoped TiO_2 and Ta:TiO_2 samples

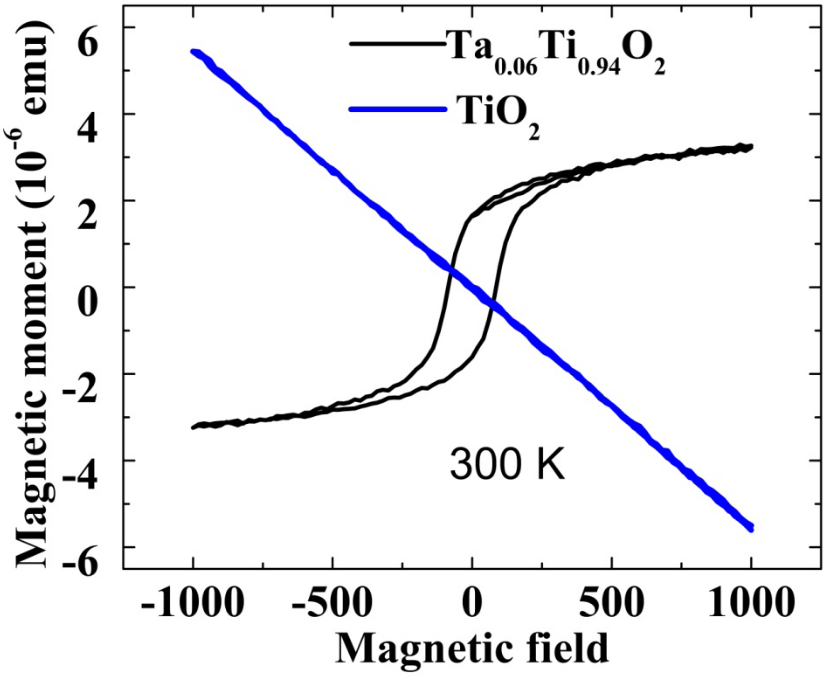

Figure S1: The above figure shows the typical room temperature in-plane M-H hysteresis loop of Ta:TiO_2 (black line) and undoped TiO_2 (blue line) grown at the same conditions on $SrTiO_3$ (001) substrate. From these results, it is clear that there is no signature of ferromagnetism either in the substrate or in undoped TiO_2.

2. Temperature dependent thermopower behavior

An independent validation for Kondo scattering is the observation of a maximum in the Seebeck coefficient close to the Kondo temperature. Figure S2 shows the Seebeck coefficient *S* as a function of temperature for 200 nm sample. The comparison between resistivity and Seebeck coefficient versus temperature indicates that as long as the resistivity is metallic (positive temperature coefficient), the Seebeck coefficient is linear in T, as predicted by Mott law:

$$S = -\frac{\pi^2}{3e} K^2 T \left.\frac{\partial \ln(\sigma(E))}{\partial E}\right|_{E=E_F} \approx -\frac{\pi^2}{3e} K^2 T \left(\left.\frac{\partial \ln(n)}{\partial E}\right|_{E=E_F} + \left.\frac{\partial \ln(\tau)}{\partial E}\right|_{E=E_F} \right) \tag{1}$$

which yields for a degenerate semiconductor in 3D:

$$S = -\left(\frac{3}{2} + \alpha\right) \frac{8\pi^{8/3} K^2}{3^{5/3} h^2 e} m_{eff} \frac{T}{n_{3D}^{2/3}} \tag{2}$$

where α describes the functional dependence of the scattering time on the energy $\tau \sim E^{\alpha}$ and its value depends on the dominant scattering mechanism. In many cases $\alpha \approx 0$ is assumed for simplicity and for lack of a solid theoretical back up, $\alpha \approx -0.5$ for scattering with acoustic phonons and for most other scattering mechanisms, α is between 0 and -1. As the carrier concentration measured by Hall effect is virtually constant as a function of temperature in our films, eq. (2) well describes the linear temperature dependence of the Seebeck curves above ~50 K, assuming effective masses around $2m_0$, where m_0 is the bare electron mass. At lower temperatures there is an onset of resistivity upturn and correspondingly also the Seebeck coefficient changes its temperature derivative sign, consistently with other literature data[1].

At lower temperatures there is an onset of resistivity upturn and correspondingly also the Seebeck coefficient changes its temperature derivative sign. In the hypothesis of Kondo regime, we can assume that below T_K a Kondo resonance yields an extra term in the Seebeck coefficient, derived from the Mott eqn. (1)[2,3]. This term can be phenomenologically expressed as :

$$S_k = -\frac{aT}{T^2 + b^2} \tag{3}$$

The coefficient ***a*** should be related to the energy position of the Kondo resonance (T_K) and to the strength of the magnetic scattering processes, while the coefficient ***b*** should be related to the energy width of the Kondo resonance. We can fit the experimental data in Fig. S2 as a sum of Mott linear Seebeck term (eq. (2)) plus a Kondo Seebeck term (eq. (3)). Associating fitting coefficients ***a*** (0.00015) and ***b*** (5) to meaningful physical parameters is not easy as the spin, charge and orbitals are coupled strongly in oxide systems.

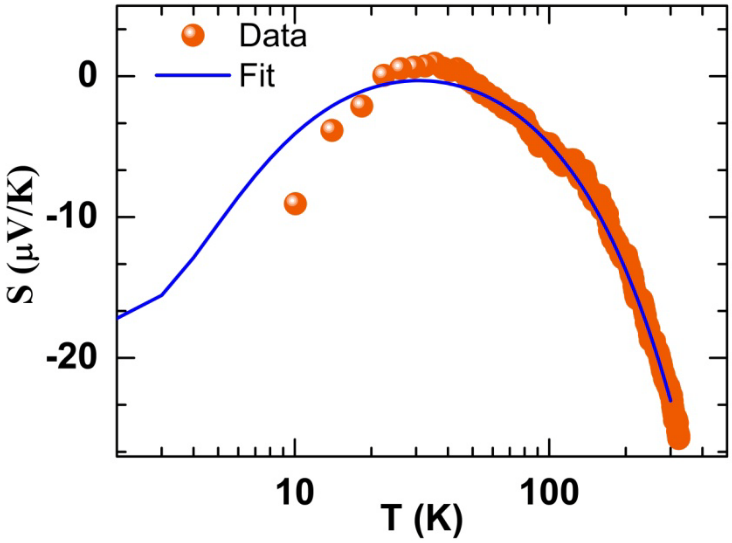

Figure S2: The thermo power as a function of temperature along with the theoretical fit.

3. Temperature dependence of the magnetic susceptibility

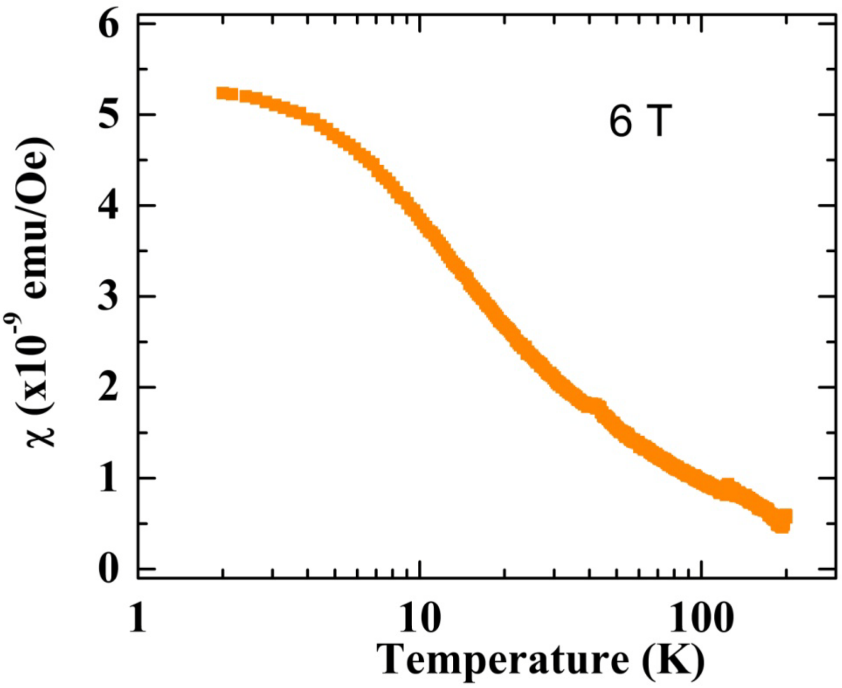

Figure S3: The temperature dependence of susceptibility (χ) for 200 nm film is shown in the temperature range from 2 to 200 K under an external magnetic field of 6 Tesla. Kondo behavior is identified from the saturation of magnetic susceptibility. However, the high temperature susceptibility could be described by Curie-Weiss $\chi = C/T\text{-}\Theta_p$ where C is the Curie consatant and Θ_p is the paramagnetic Curie temperature. Figure S3 shows the

susceptibility below ~6 K tends to saturate which deviates from the Curie-Weiss law. This deviation is similar to that reported by other groups[4] which support our Kondo model .

4. Photoluminescence spectra for different concentrations of Ta in TiO_2

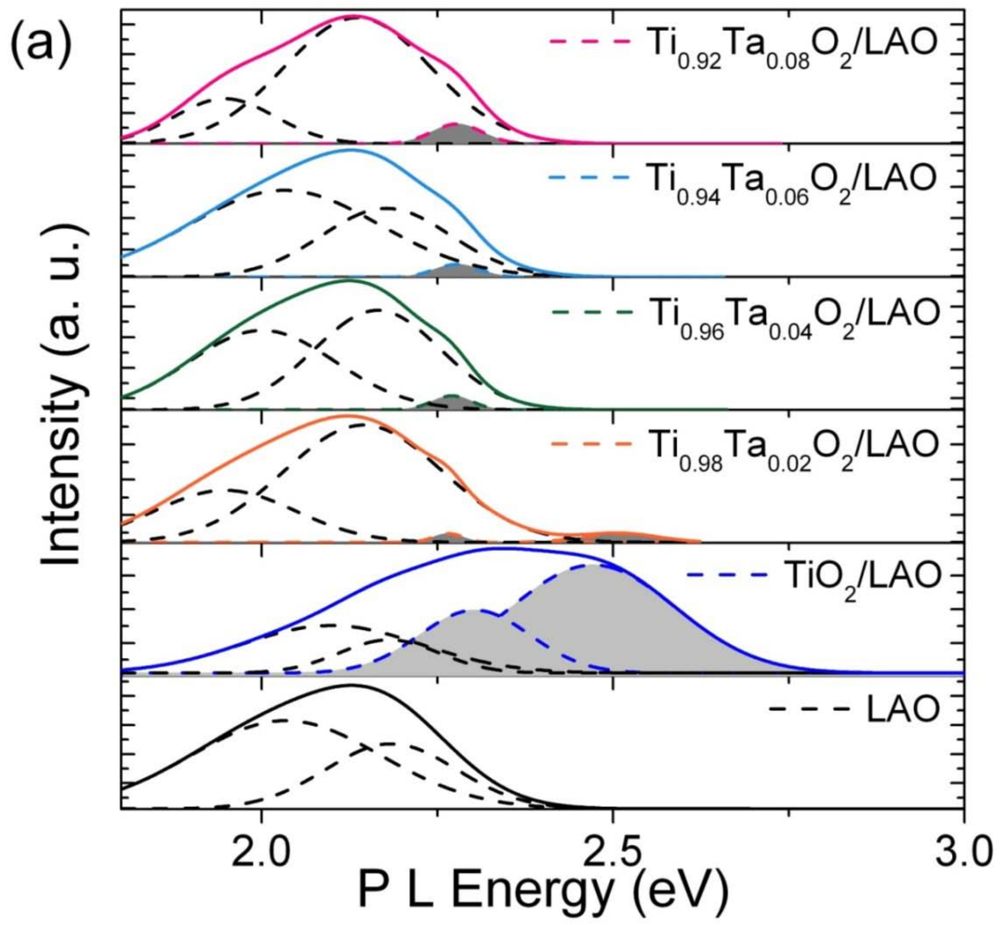

Figure S4: The normalised PL spectra obtained at 20 K for $Ti_{1-x}Ta_xO_2$ thin films for different Ta concentrations (0 to 8%) at the low energy range (1.8 eV to 3.0 eV). We observed the Stoke-shifted self-trapped exciton peak centered at 2.35 eV for the undoped TiO_2 film. This is in accordance with the previous PL studies done on TiO_2. The origin of this broad peak has been assigned to oxygen vacancies in the TiO_2 crystal which form defect levels deep inside the bandgap. The broad PL peak for undoped TiO_2 thin films was deconvoluted into four Gaussian peaks centered at around 2.05, 2.2, 2.3 and 2.5 eV. Next, the PL spectra from a bare LAO substrate (treated under the same conditions under which the films were grown) were obtained. It was found out that LAO itself has a broad PL peak, which in turn is deconvoluted into two Gaussian components. These two Gaussians matched

pretty closely with the 2.05 and 2.2 eV peaks for the undoped TiO_2 (Figure S4). Hence, we can safely assign these two lower energy peaks to the oxygen vacancies in the LAO substrates. As a result, only the other two peaks at 2.3 and 2.5 eV can then be argued to be coming from TiO_2. We further observed that the Gaussians at 2.3 and 2.5 eV (due to TiO_2) were sharply quenched on the slightest Ta incorporation for the $Ti_{1-x}Ta_xO_2$ thin films. It may be concluded here that the signal obtained for the $Ti_{1-x}Ta_xO_2$ thin films were actually due to the LAO substrate. Ta incorporation in TiO_2 enhances free donor electrons in the system. Consequently, the self compensating nature of the crystal acts to enhance the formation energy for any electron donor defects in the system. This explains the quenching of the PL peaks (arising due to oxygen vacancies) in $Ti_{1-x}Ta_xO_2$ thin films. A new peak is found to increase in intensity with Ta concentration at 2.27 eV which may be related to cationic defects which tend to increase with Ta concentration.

References:

1 Forro, L., Chauvet, O., Emin, D., Zuppiroli, L., Berger, H. & Lévy, F. High mobility n -type charge carriers in large single crystals of anatase (TiO_2). *J. Appl. Phys.* **75**, 633-635 (1994).

2 Koterlyn, M. D., Babych, O. I. & Koterlyn, G. M. Dependence of the $CeNi_5$ thermoelectric power on strong 4f-electron instability. *J. Alloys Compd.* **325**, 6-11 (2001).

3 Gofryk, K., Griveau, J. C., Colineau, E., Sanchez, J. P., Rebizant, J. & Caciuffo, R. Kondo behavior in superconducting $NpPd5Al_2$. *Phys. Rev. B* **79**, 134525 (2009).

4 Li, L., Yamagata, R., Nishimura, K. & Yamaoka, H. Structure, magnetic ordering, and Kondo effect in $(Ce_{1-x}Nd_x)_3Al$. *Phys. Rev. B* **80**, 134429 (2009).
